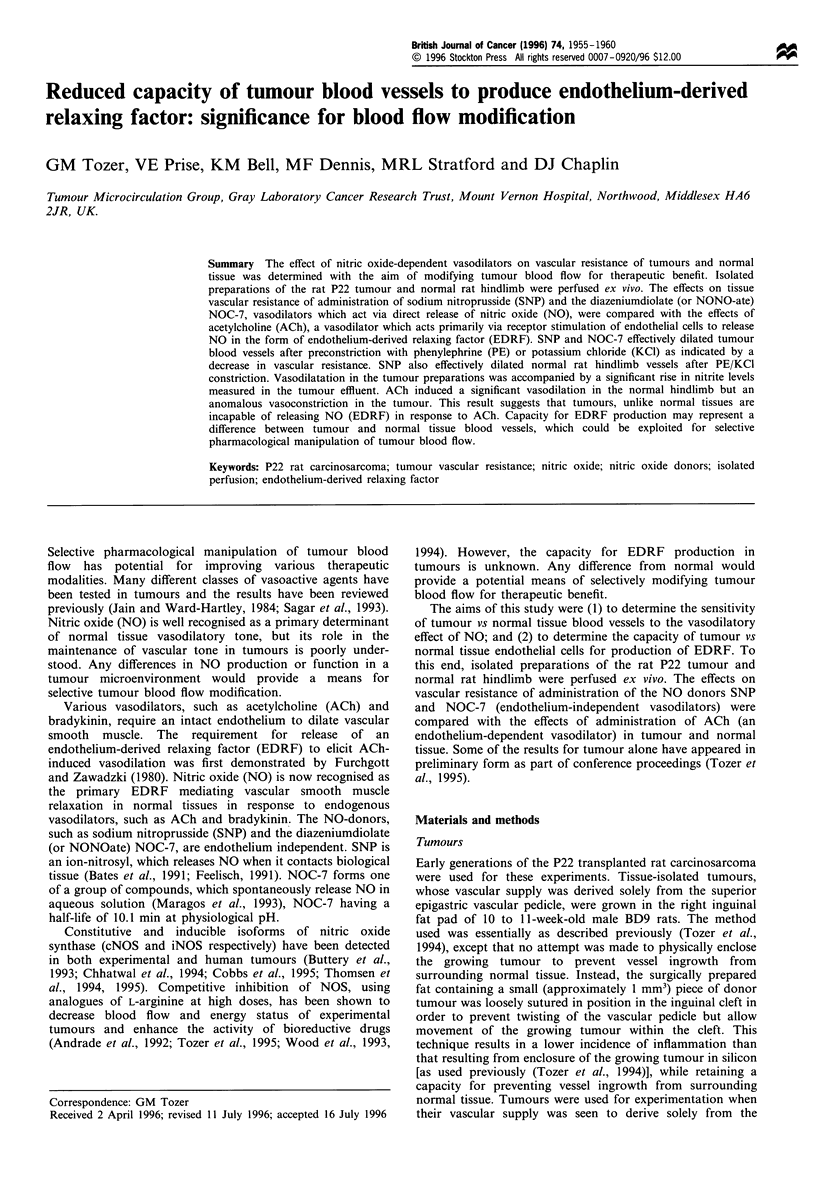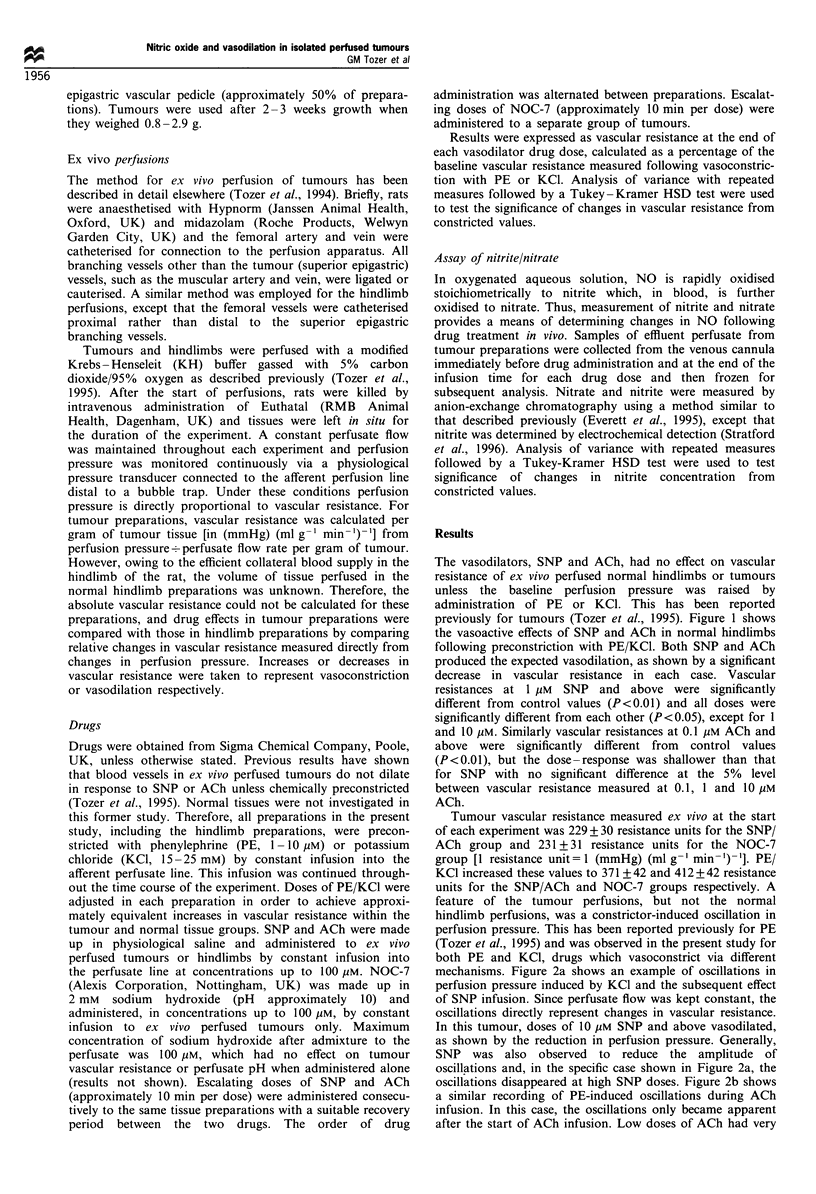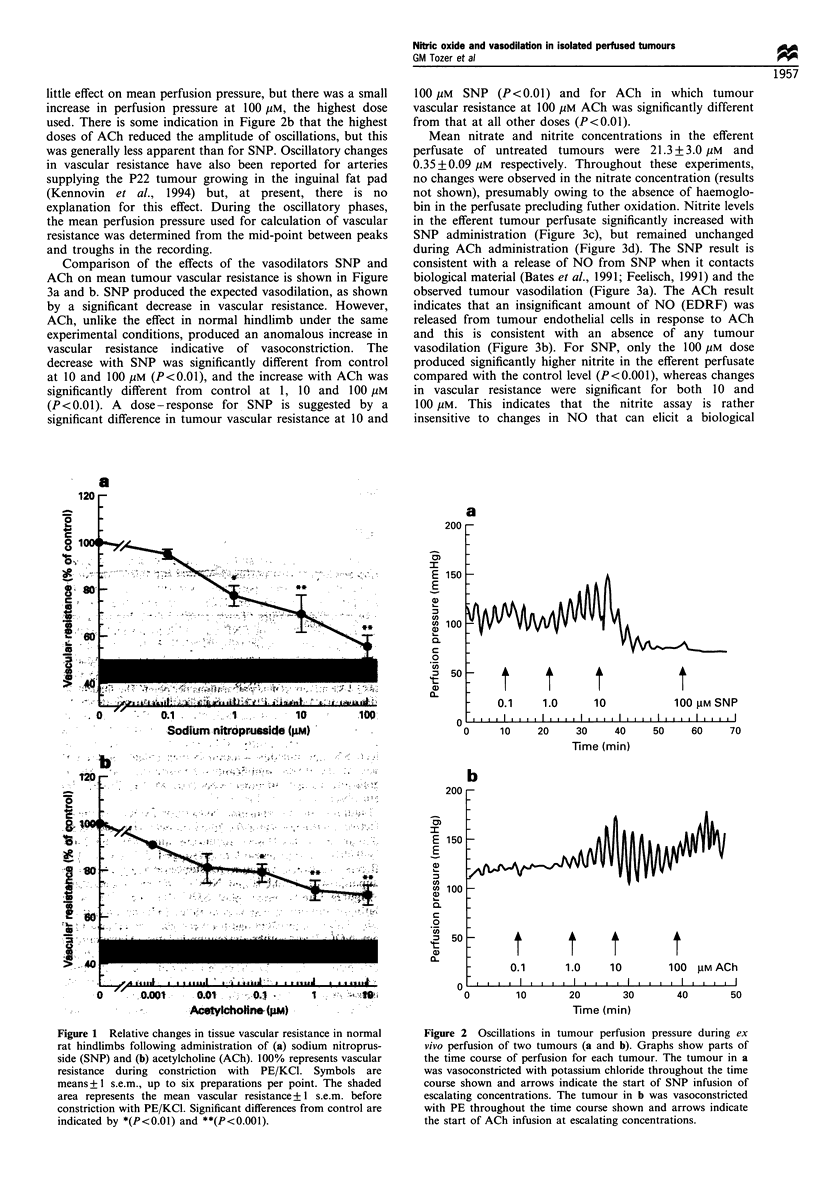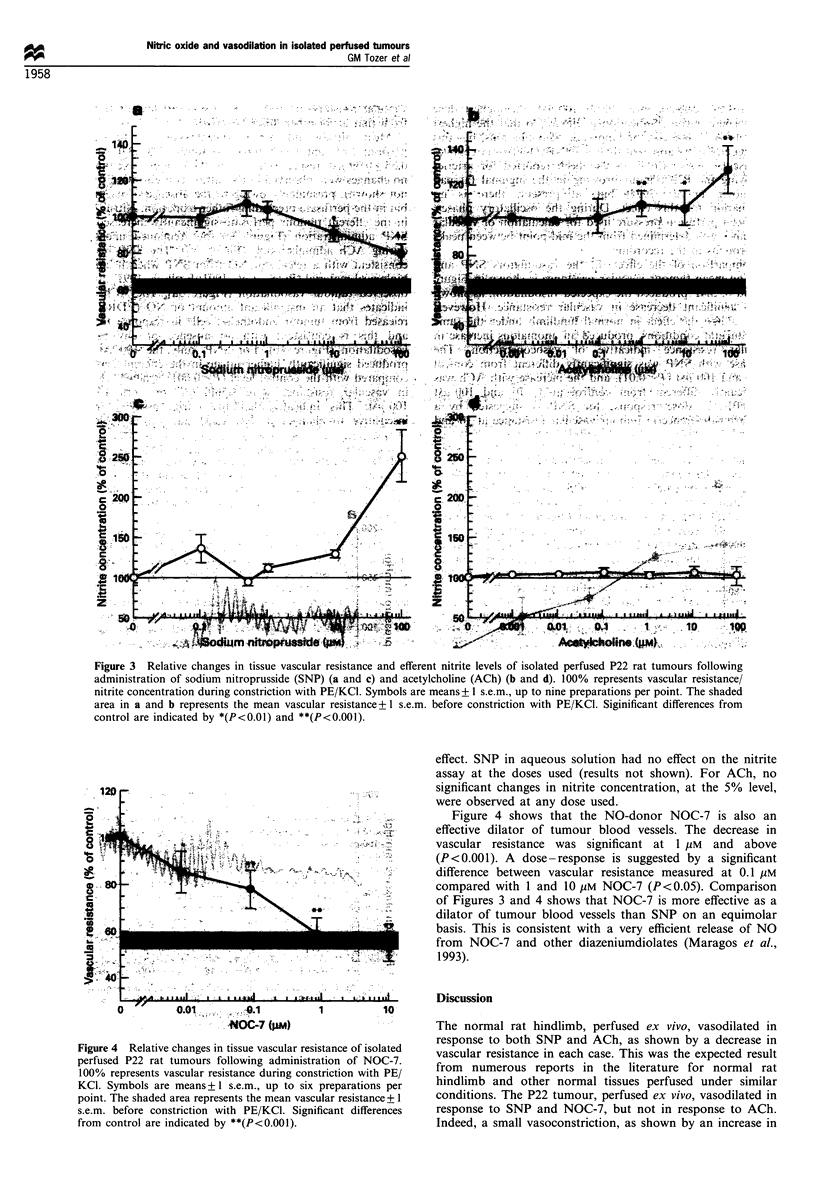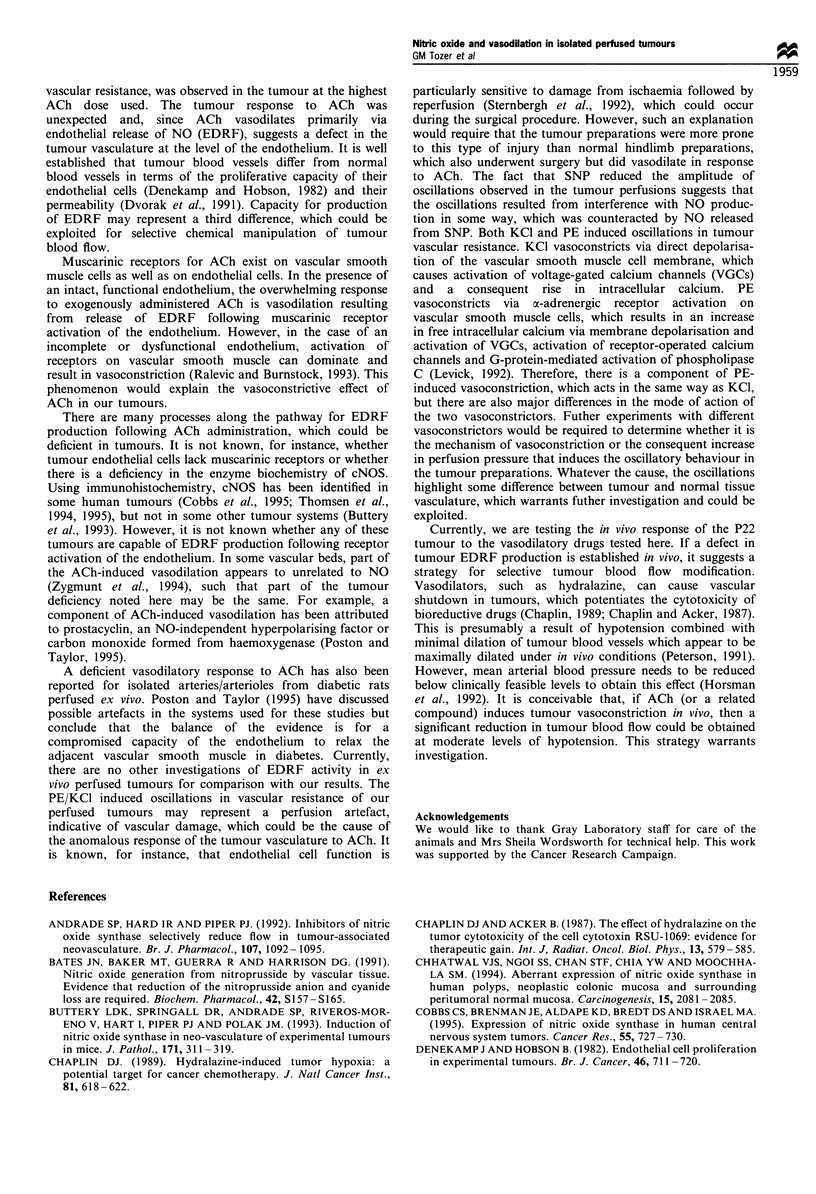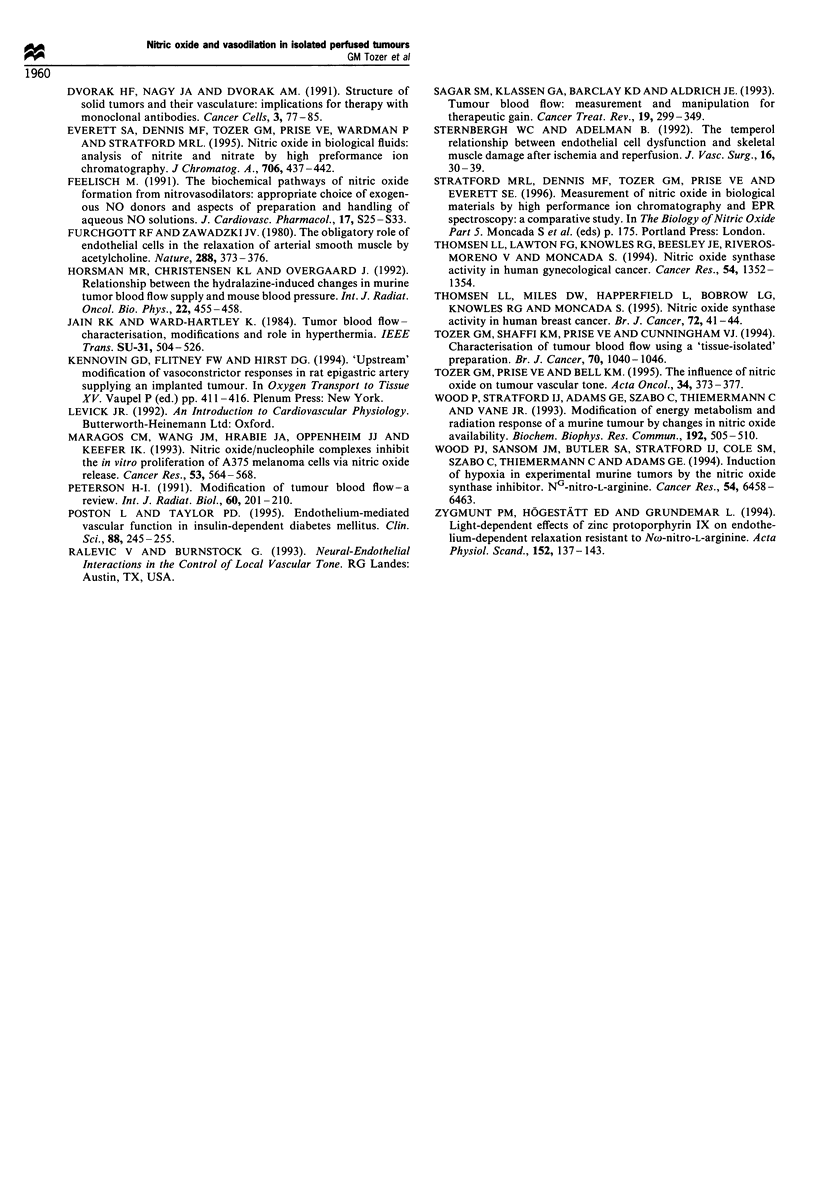# Reduced capacity of tumour blood vessels to produce endothelium-derived relaxing factor: significance for blood flow modification.

**DOI:** 10.1038/bjc.1996.659

**Published:** 1996-12

**Authors:** G. M. Tozer, V. E. Prise, K. M. Bell, M. F. Dennis, M. R. Stratford, D. J. Chaplin

**Affiliations:** Gray Laboratory Cancer Research Trust, Mount Vernon Hospital, Northwood, Middlesex, UK.

## Abstract

The effect of nitric oxide-dependent vasodilators on vascular resistance of tumours and normal tissue was determined with the aim of modifying tumour blood flow for therapeutic benefit. Isolated preparations of the rat P22 tumour and normal rat hindlimb were perfused ex vivo. The effects on tissue vascular resistance of administration of sodium nitroprusside (SNP) and the diazeniumdiolate (or NONO-ate) NOC-7, vasodilators which act via direct release of nitric oxide (NO), were compared with the effects of acetylcholine (ACh), a vasodilator which acts primarily via receptor stimulation of endothelial cells to release NO in the form of endothelium-derived relaxing factor (EDRF). SNP and NOC-7 effectively dilated tumour blood vessels after preconstriction with phenylephrine (PE) or potassium chloride (KCl) as indicated by a decrease in vascular resistance. SNP also effectively dilated normal rat hindlimb vessels after PE/KCl constriction. Vasodilatation in the tumour preparations was accompanied by a significant rise in nitrite levels measured in the tumour effluent. ACh induced a significant vasodilation in the normal hindlimb but an anomalous vasoconstriction in the tumour. This result suggests that tumours, unlike normal tissues are incapable of releasing NO (EDRF) in response to ACh. Capacity for EDRF production may represent a difference between tumour and normal tissue blood vessels, which could be exploited for selective pharmacological manipulation of tumour blood flow.


British Journal of Cancer (1996) 74, 1955-1960
© 1996 Stockton Press All rights reserved 0007-0920/96 $12.00

### Reduced capacity of tumour blood vessels to produce endothelium-derived relaxing factor: significance for blood flow modification

GM Tozer, VE Prise, KM Bell, MF Dennis, MRL Stratford and DJ Chaplin

*Tumour Microcirculation Group, Gray Laboratory Cancer Research Trust, Mount Vernon Hospital, Northwood, Middlesex HA6 2JR, UK.*

**Summary** The effect of nitric oxide-dependent vasodilators on vascular resistance of tumours and normal tissue was determined with the aim of modifying tumour blood flow for therapeutic benefit. Isolated preparations of the rat P22 tumour and normal rat hindlimb were perfused *ex vivo*. The effects on tissue vascular resistance of administration of sodium nitroprusside (SNP) and the diazeniumdiolate (or NONO-ate) NOC-7, vasodilators which act via direct release of nitric oxide (NO), were compared with the effects of acetylcholine (ACh), a vasodilator which acts primarily via receptor stimulation of endothelial cells to release NO in the form of endothelium-derived relaxing factor (EDRF). SNP and NOC-7 effectively dilated tumour blood vessels after preconstriction with phenylephrine (PE) or potassium chloride (KCl) as indicated by a decrease in vascular resistance. SNP also effectively dilated normal rat hindlimb vessels after PE/KCl constriction. Vasodilatation in the tumour preparations was accompanied by a significant rise in nitrite levels measured in the tumour effluent. ACh induced a significant vasodilation in the normal hindlimb but an anomalous vasoconstriction in the tumour. This result suggests that tumours, unlike normal tissues are incapable of releasing NO (EDRF) in response to ACh. Capacity for EDRF production may represent a difference between tumour and normal tissue blood vessels, which could be exploited for selective pharmacological manipulation of tumour blood flow.

**Keywords:** P22 rat carcinosarcoma; tumour vascular resistance; nitric oxide; nitric oxide donors; isolated perfusion; endothelium-derived relaxing factor

Selective pharmacological manipulation of tumour blood flow has potential for improving various therapeutic modalities. Many different classes of vasoactive agents have been tested in tumours and the results have been reviewed previously (Jain and Ward-Hartley, 1984; Sagar *et al.*, 1993). Nitric oxide (NO) is well recognised as a primary determinant of normal tissue vasodilatory tone, but its role in the maintenance of vascular tone in tumours is poorly understood. Any differences in NO production or function in a tumour microenvironment would provide a means for selective tumour blood flow modification.

Various vasodilators, such as acetylcholine (ACh) and bradykinin, require an intact endothelium to dilate vascular smooth muscle. The requirement for release of an endothelium-derived relaxing factor (EDRF) to elicit ACh-induced vasodilation was first demonstrated by Furchgott and Zawadzki (1980). Nitric oxide (NO) is now recognised as the primary EDRF mediating vascular smooth muscle relaxation in normal tissues in response to endogenous vasodilators, such as ACh and bradykinin. The NO-donors, such as sodium nitroprusside (SNP) and the diazeniumdiolate (or NONOate) NOC-7, are endothelium independent. SNP is an ion-nitrosyl, which releases NO when it contacts biological tissue (Bates *et al.*, 1991; Feelisch, 1991). NOC-7 forms one of a group of compounds, which spontaneously release NO in aqueous solution (Maragos *et al.*, 1993), NOC-7 having a half-life of 10.1 min at physiological pH.

Constitutive and inducible isoforms of nitric oxide synthase (cNOS and iNOS respectively) have been detected in both experimental and human tumours (Buttery *et al.*, 1993; Chhatwal *et al.*, 1994; Cobbs *et al.*, 1995; Thomsen *et al.*, 1994, 1995). Competitive inhibition of NOS, using analogues of L-arginine at high doses, has been shown to decrease blood flow and energy status of experimental tumours and enhance the activity of bioreductive drugs (Andrade *et al.*, 1992; Tozer *et al.*, 1995; Wood *et al.*, 1993, 1994). However, the capacity for EDRF production in tumours is unknown. Any difference from normal would provide a potential means of selectively modifying tumour blood flow for therapeutic benefit.

The aims of this study were (1) to determine the sensitivity of tumour *vs* normal tissue blood vessels to the vasodilatory effect of NO; and (2) to determine the capacity of tumour *vs* normal tissue endothelial cells for production of EDRF. To this end, isolated preparations of the rat P22 tumour and normal rat hindlimb were perfused *ex vivo*. The effects on vascular resistance of administration of the NO donors SNP and NOC-7 (endothelium-independent vasodilators) were compared with the effects of administration of ACh (an endothelium-dependent vasodilator) in tumour and normal tissue. Some of the results for tumour alone have appeared in preliminary form as part of conference proceedings (Tozer *et al.*, 1995).

### Materials and methods

### *Tumours*

Early generations of the P22 transplanted rat carcinosarcoma were used for these experiments. Tissue-isolated tumours, whose vascular supply was derived solely from the superior epigastric vascular pedicle, were grown in the right inguinal fat pad of 10 to 11-week-old male BD9 rats. The method used was essentially as described previously (Tozer *et al.*, 1994), except that no attempt was made to physically enclose the growing tumour to prevent vessel ingrowth from surrounding normal tissue. Instead, the surgically prepared fat containing a small (approximately 1 $mm^3$) piece of donor tumour was loosely sutured in position in the inguinal cleft in order to prevent twisting of the vascular pedicle but allow movement of the growing tumour within the cleft. This technique results in a lower incidence of inflammation than that resulting from enclosure of the growing tumour in silicon [as used previously (Tozer *et al.*, 1994)], while retaining a capacity for preventing vessel ingrowth from surrounding normal tissue. Tumours were used for experimentation when their vascular supply was seen to derive solely from the

Correspondence: GM Tozer
Received 2 April 1996; revised 11 July 1996; accepted 16 July 1996

epigastric vascular pedicle (approximately 50% of preparations). Tumours were used after 2–3 weeks growth when they weighed 0.8–2.9 g.

### Ex vivo *perfusions*

The method for *ex vivo* perfusion of tumours has been described in detail elsewhere (Tozer *et al.*, 1994). Briefly, rats were anaesthetised with Hypnorm (Janssen Animal Health, Oxford, UK) and midazolam (Roche Products, Welwyn Garden City, UK) and the femoral artery and vein were catheterised for connection to the perfusion apparatus. All branching vessels other than the tumour (superior epigastric) vessels, such as the muscular artery and vein, were ligated or cauterised. A similar method was employed for the hindlimb perfusions, except that the femoral vessels were catheterised proximal rather than distal to the superior epigastric branching vessels.

Tumours and hindlimbs were perfused with a modified Krebs–Henseleit (KH) buffer gassed with 5% carbon dioxide/95% oxygen as described previously (Tozer *et al.*, 1995). After the start of perfusions, rats were killed by intravenous administration of Euthatal (RMB Animal Health, Dagenham, UK) and tissues were left *in situ* for the duration of the experiment. A constant perfusate flow was maintained throughout each experiment and perfusion pressure was monitored continuously via a physiological pressure transducer connected to the afferent perfusion line distal to a bubble trap. Under these conditions perfusion pressure is directly proportional to vascular resistance. For tumour preparations, vascular resistance was calculated per gram of tumour tissue [in (mmHg) $(\text{ml g}^{-1}\ \text{min}^{-1})^{-1}$] from perfusion pressure ÷ perfusate flow rate per gram of tumour. However, owing to the efficient collateral blood supply in the hindlimb of the rat, the volume of tissue perfused in the normal hindlimb preparations was unknown. Therefore, the absolute vascular resistance could not be calculated for these preparations, and drug effects in tumour preparations were compared with those in hindlimb preparations by comparing relative changes in vascular resistance measured directly from changes in perfusion pressure. Increases or decreases in vascular resistance were taken to represent vasoconstriction or vasodilation respectively.

### *Drugs*

Drugs were obtained from Sigma Chemical Company, Poole, UK, unless otherwise stated. Previous results have shown that blood vessels in *ex vivo* perfused tumours do not dilate in response to SNP or ACh unless chemically preconstricted (Tozer *et al.*, 1995). Normal tissues were not investigated in this former study. Therefore, all preparations in the present study, including the hindlimb preparations, were preconstricted with phenylephrine (PE, 1–10 μM) or potassium chloride (KCl, 15–25 mM) by constant infusion into the afferent perfusate line. This infusion was continued throughout the time course of the experiment. Doses of PE/KCl were adjusted in each preparation in order to achieve approximately equivalent increases in vascular resistance within the tumour and normal tissue groups. SNP and ACh were made up in physiological saline and administered to *ex vivo* perfused tumours or hindlimbs by constant infusion into the perfusate line at concentrations up to 100 μM. NOC-7 (Alexis Corporation, Nottingham, UK) was made up in 2 mM sodium hydroxide (pH approximately 10) and administered, in concentrations up to 100 μM, by constant infusion to *ex vivo* perfused tumours only. Maximum concentration of sodium hydroxide after admixture to the perfusate was 100 μM, which had no effect on tumour vascular resistance or perfusate pH when administered alone (results not shown). Escalating doses of SNP and ACh (approximately 10 min per dose) were administered consecutively to the same tissue preparations with a suitable recovery period between the two drugs. The order of drug administration was alternated between preparations. Escalating doses of NOC-7 (approximately 10 min per dose) were administered to a separate group of tumours.

Results were expressed as vascular resistance at the end of each vasodilator drug dose, calculated as a percentage of the baseline vascular resistance measured following vasoconstriction with PE or KCl. Analysis of variance with repeated measures followed by a Tukey–Kramer HSD test were used to test the significance of changes in vascular resistance from constricted values.

### *Assay of nitrite/nitrate*

In oxygenated aqueous solution, NO is rapidly oxidised stoichiometrically to nitrite which, in blood, is further oxidised to nitrate. Thus, measurement of nitrite and nitrate provides a means of determining changes in NO following drug treatment *in vivo*. Samples of effluent perfusate from tumour preparations were collected from the venous cannula immediately before drug administration and at the end of the infusion time for each drug dose and then frozen for subsequent analysis. Nitrate and nitrite were measured by anion-exchange chromatography using a method similar to that described previously (Everett *et al.*, 1995), except that nitrite was determined by electrochemical detection (Stratford *et al.*, 1996). Analysis of variance with repeated measures followed by a Tukey-Kramer HSD test were used to test significance of changes in nitrite concentration from constricted values.

### Results

The vasodilators, SNP and ACh, had no effect on vascular resistance of *ex vivo* perfused normal hindlimbs or tumours unless the baseline perfusion pressure was raised by administration of PE or KCl. This has been reported previously for tumours (Tozer *et al.*, 1995). Figure 1 shows the vasoactive effects of SNP and ACh in normal hindlimbs following preconstriction with PE/KCl. Both SNP and ACh produced the expected vasodilation, as shown by a significant decrease in vascular resistance in each case. Vascular resistances at 1 μM SNP and above were significantly different from control values ($P<0.01$) and all doses were significantly different from each other ($P<0.05$), except for 1 and 10 μM. Similarly vascular resistances at 0.1 μM ACh and above were significantly different from control values ($P<0.01$), but the dose–response was shallower than that for SNP with no significant difference at the 5% level between vascular resistance measured at 0.1, 1 and 10 μM ACh.

Tumour vascular resistance measured *ex vivo* at the start of each experiment was $229 \pm 30$ resistance units for the SNP/ACh group and $231 \pm 31$ resistance units for the NOC-7 group [1 resistance unit = 1 (mmHg) $(\text{ml g}^{-1}\ \text{min}^{-1})^{-1}$]. PE/KCl increased these values to $371 \pm 42$ and $412 \pm 42$ resistance units for the SNP/ACh and NOC-7 groups respectively. A feature of the tumour perfusions, but not the normal hindlimb perfusions, was a constrictor-induced oscillation in perfusion pressure. This has been reported previously for PE (Tozer *et al.*, 1995) and was observed in the present study for both PE and KCl, drugs which vasoconstrict via different mechanisms. Figure 2a shows an example of oscillations in perfusion pressure induced by KCl and the subsequent effect of SNP infusion. Since perfusate flow was kept constant, the oscillations directly represent changes in vascular resistance. In this tumour, doses of 10 μM SNP and above vasodilated, as shown by the reduction in perfusion pressure. Generally, SNP was also observed to reduce the amplitude of oscillations and, in the specific case shown in Figure 2a, the oscillations disappeared at high SNP doses. Figure 2b shows a similar recording of PE-induced oscillations during ACh infusion. In this case, the oscillations only became apparent after the start of ACh infusion. Low doses of ACh had very little effect on mean perfusion pressure, but there was a small increase in perfusion pressure at 100 μM, the highest dose used. There is some indication in Figure 2b that the highest doses of ACh reduced the amplitude of oscillations, but this was generally less apparent than for SNP. Oscillatory changes in vascular resistance have also been reported for arteries supplying the P22 tumour growing in the inguinal fat pad (Kennovin *et al.*, 1994) but, at present, there is no explanation for this effect. During the oscillatory phases, the mean perfusion pressure used for calculation of vascular resistance was determined from the mid-point between peaks and troughs in the recording.

Comparison of the effects of the vasodilators SNP and ACh on mean tumour vascular resistance is shown in Figure 3a and b. SNP produced the expected vasodilation, as shown by a significant decrease in vascular resistance. However, ACh, unlike the effect in normal hindlimb under the same experimental conditions, produced an anomalous increase in vascular resistance indicative of vasoconstriction. The decrease with SNP was significantly different from control at 10 and 100 μM ($P<0.01$), and the increase with ACh was significantly different from control at 1, 10 and 100 μM ($P<0.01$). A dose-response for SNP is suggested by a significant difference in tumour vascular resistance at 10 and 100 μM SNP ($P<0.01$) and for ACh in which tumour vascular resistance at 100 μM ACh was significantly different from that at all other doses ($P<0.01$).

Mean nitrate and nitrite concentrations in the efferent perfusate of untreated tumours were $21.3 \pm 3.0$ μM and $0.35 \pm 0.09$ μM respectively. Throughout these experiments, no changes were observed in the nitrate concentration (results not shown), presumably owing to the absence of haemoglobin in the perfusate precluding futher oxidation. Nitrite levels in the efferent tumour perfusate significantly increased with SNP administration (Figure 3c), but remained unchanged during ACh administration (Figure 3d). The SNP result is consistent with a release of NO from SNP when it contacts biological material (Bates *et al.*, 1991; Feelisch, 1991) and the observed tumour vasodilation (Figure 3a). The ACh result indicates that an insignificant amount of NO (EDRF) was released from tumour endothelial cells in response to ACh and this is consistent with an absence of any tumour vasodilation (Figure 3b). For SNP, only the 100 μM dose produced significantly higher nitrite in the efferent perfusate compared with the control level ($P<0.001$), whereas changes in vascular resistance were significant for both 10 and 100 μM. This indicates that the nitrite assay is rather insensitive to changes in NO that can elicit a biological

**Figure 1** Relative changes in tissue vascular resistance in normal rat hindlimbs following administration of (**a**) sodium nitroprusside (SNP) and (**b**) acetylcholine (ACh). 100% represents vascular resistance during constriction with PE/KCl. Symbols are means±1 s.e.m., up to six preparations per point. The shaded area represents the mean vascular resistance±1 s.e.m. before constriction with PE/KCl. Significant differences from control are indicated by *($P<0.01$) and **($P<0.001$).

**Figure 2** Oscillations in tumour perfusion pressure during *ex vivo* perfusion of two tumours (**a** and **b**). Graphs show parts of the time course of perfusion for each tumour. The tumour in **a** was vasoconstricted with potassium chloride throughout the time course shown and arrows indicate the start of SNP infusion of escalating concentrations. The tumour in **b** was vasoconstricted with PE throughout the time course shown and arrows indicate the start of ACh infusion at escalating concentrations.

**Figure 3** Relative changes in tissue vascular resistance and efferent nitrite levels of isolated perfused P22 rat tumours following administration of sodium nitroprusside (SNP) (**a** and **c**) and acetylcholine (ACh) (**b** and **d**). 100% represents vascular resistance/nitrite concentration during constriction with PE/KCl. Symbols are means ± 1 s.e.m., up to nine preparations per point. The shaded area in **a** and **b** represents the mean vascular resistance ± 1 s.e.m. before constriction with PE/KCl. Siginificant differences from control are indicated by *($P<0.01$) and **($P<0.001$).

**Figure 4** Relative changes in tissue vascular resistance of isolated perfused P22 rat tumours following administration of NOC-7. 100% represents vascular resistance during constriction with PE/KCl. Symbols are means ± 1 s.e.m., up to six preparations per point. The shaded area represents the mean vascular resistance ± 1 s.e.m. before constriction with PE/KCl. Significant differences from control are indicated by **($P<0.001$).

effect. SNP in aqueous solution had no effect on the nitrite assay at the doses used (results not shown). For ACh, no significant changes in nitrite concentration, at the 5% level, were observed at any dose used.

Figure 4 shows that the NO-donor NOC-7 is also an effective dilator of tumour blood vessels. The decrease in vascular resistance was significant at 1 μM and above ($P<0.001$). A dose–response is suggested by a significant difference between vascular resistance measured at 0.1 μM compared with 1 and 10 μM NOC-7 ($P<0.05$). Comparison of Figures 3 and 4 shows that NOC-7 is more effective as a dilator of tumour blood vessels than SNP on an equimolar basis. This is consistent with a very efficient release of NO from NOC-7 and other diazeniumdiolates (Maragos *et al.*, 1993).

### Discussion

The normal rat hindlimb, perfused *ex vivo*, vasodilated in response to both SNP and ACh, as shown by a decrease in vascular resistance in each case. This was the expected result from numerous reports in the literature for normal rat hindlimb and other normal tissues perfused under similar conditions. The P22 tumour, perfused *ex vivo*, vasodilated in response to SNP and NOC-7, but not in response to ACh. Indeed, a small vasoconstriction, as shown by an increase in vascular resistance, was observed in the tumour at the highest ACh dose used. The tumour response to ACh was unexpected and, since ACh vasodilates primarily via endothelial release of NO (EDRF), suggests a defect in the tumour vasculature at the level of the endothelium. It is well established that tumour blood vessels differ from normal blood vessels in terms of the proliferative capacity of their endothelial cells (Denekamp and Hobson, 1982) and their permeability (Dvorak *et al.*, 1991). Capacity for production of EDRF may represent a third difference, which could be exploited for selective chemical manipulation of tumour blood flow.

Muscarinic receptors for ACh exist on vascular smooth muscle cells as well as on endothelial cells. In the presence of an intact, functional endothelium, the overwhelming response to exogenously administered ACh is vasodilation resulting from release of EDRF following muscarinic receptor activation of the endothelium. However, in the case of an incomplete or dysfunctional endothelium, activation of receptors on vascular smooth muscle can dominate and result in vasoconstriction (Ralevic and Burnstock, 1993). This phenomenon would explain the vasoconstrictive effect of ACh in our tumours.

There are many processes along the pathway for EDRF production following ACh administration, which could be deficient in tumours. It is not known, for instance, whether tumour endothelial cells lack muscarinic receptors or whether there is a deficiency in the enzyme biochemistry of cNOS. Using immunohistochemistry, cNOS has been identified in some human tumours (Cobbs *et al.*, 1995; Thomsen *et al.*, 1994, 1995), but not in some other tumour systems (Buttery *et al.*, 1993). However, it is not known whether any of these tumours are capable of EDRF production following receptor activation of the endothelium. In some vascular beds, part of the ACh-induced vasodilation appears to unrelated to NO (Zygmunt *et al.*, 1994), such that part of the tumour deficiency noted here may be the same. For example, a component of ACh-induced vasodilation has been attributed to prostacyclin, an NO-independent hyperpolarising factor or carbon monoxide formed from haemoxygenase (Poston and Taylor, 1995).

A deficient vasodilatory response to ACh has also been reported for isolated arteries/arterioles from diabetic rats perfused *ex vivo*. Poston and Taylor (1995) have discussed possible artefacts in the systems used for these studies but conclude that the balance of the evidence is for a compromised capacity of the endothelium to relax the adjacent vascular smooth muscle in diabetes. Currently, there are no other investigations of EDRF activity in *ex vivo* perfused tumours for comparison with our results. The PE/KCl induced oscillations in vascular resistance of our perfused tumours may represent a perfusion artefact, indicative of vascular damage, which could be the cause of the anomalous response of the tumour vasculature to ACh. It is known, for instance, that endothelial cell function is particularly sensitive to damage from ischaemia followed by reperfusion (Sternbergh *et al.*, 1992), which could occur during the surgical procedure. However, such an explanation would require that the tumour preparations were more prone to this type of injury than normal hindlimb preparations, which also underwent surgery but did vasodilate in response to ACh. The fact that SNP reduced the amplitude of oscillations observed in the tumour perfusions suggests that the oscillations resulted from interference with NO production in some way, which was counteracted by NO released from SNP. Both KCl and PE induced oscillations in tumour vascular resistance. KCl vasoconstricts via direct depolarisation of the vascular smooth muscle cell membrane, which causes activation of voltage-gated calcium channels (VGCs) and a consequent rise in intracellular calcium. PE vasoconstricts via α-adrenergic receptor activation on vascular smooth muscle cells, which results in an increase in free intracellular calcium via membrane depolarisation and activation of VGCs, activation of receptor-operated calcium channels and G-protein-mediated activation of phospholipase C (Levick, 1992). Therefore, there is a component of PE-induced vasoconstriction, which acts in the same way as KCl, but there are also major differences in the mode of action of the two vasoconstrictors. Futher experiments with different vasoconstrictors would be required to determine whether it is the mechanism of vasoconstriction or the consequent increase in perfusion pressure that induces the oscillatory behaviour in the tumour preparations. Whatever the cause, the oscillations highlight some difference between tumour and normal tissue vasculature, which warrants futher investigation and could be exploited.

Currently, we are testing the *in vivo* response of the P22 tumour to the vasodilatory drugs tested here. If a defect in tumour EDRF production is established *in vivo*, it suggests a strategy for selective tumour blood flow modification. Vasodilators, such as hydralazine, can cause vascular shutdown in tumours, which potentiates the cytotoxicity of bioreductive drugs (Chaplin, 1989; Chaplin and Acker, 1987). This is presumably a result of hypotension combined with minimal dilation of tumour blood vessels which appear to be maximally dilated under *in vivo* conditions (Peterson, 1991). However, mean arterial blood pressure needs to be reduced below clinically feasible levels to obtain this effect (Horsman *et al.*, 1992). It is conceivable that, if ACh (or a related compound) induces tumour vasoconstriction *in vivo*, then a significant reduction in tumour blood flow could be obtained at moderate levels of hypotension. This strategy warrants investigation.

**Acknowledgements**
We would like to thank Gray Laboratory staff for care of the animals and Mrs Sheila Wordsworth for technical help. This work was supported by the Cancer Research Campaign.

### References

ANDRADE SP, HARD IR AND PIPER PJ. (1992). Inhibitors of nitric oxide synthase selectively reduce flow in tumour-associated neovasculature. *Br. J. Pharmacol.*, **107**, 1092–1095.

BATES JN, BAKER MT, GUERRA R AND HARRISON DG. (1991). Nitric oxide generation from nitroprusside by vascular tissue. Evidence that reduction of the nitroprusside anion and cyanide loss are required. *Biochem. Pharmacol.*, **42**, S157–S165.

BUTTERY LDK, SPRINGALL DR, ANDRADE SP, RIVEROS-MORENO V, HART I, PIPER PJ AND POLAK JM. (1993). Induction of nitric oxide synthase in neo-vasculature of experimental tumours in mice. *J. Pathol.*, **171**, 311–319.

CHAPLIN DJ. (1989). Hydralazine-induced tumor hypoxia: a potential target for cancer chemotherapy. *J. Natl Cancer Inst.*, **81**, 618–622.

CHAPLIN DJ AND ACKER B. (1987). The effect of hydralazine on the tumor cytotoxicity of the cell cytotoxin RSU-1069: evidence for therapeutic gain. *Int. J, Radiat. Oncol. Biol. Phys.*, **13**, 579–585.

CHHATWAL VJS, NGOI SS, CHAN STF, CHIA YW AND MOOCHHALA SM. (1994). Aberrant expression of nitric oxide synthase in human polyps, neoplastic colonic mucosa and surrounding peritumoral normal mucosa. *Carcinogenesis*, **15**, 2081–2085.

COBBS CS, BRENMAN JE, ALDAPE KD, BREDT DS AND ISRAEL MA. (1995). Expression of nitric oxide synthase in human central nervous system tumors. *Cancer Res.*, **55**, 727–730.

DENEKAMP J AND HOBSON B. (1982). Endothelial cell proliferation in experimental tumours. *Br. J. Cancer*, **46**, 711–720.

DVORAK HF, NAGY JA AND DVORAK AM. (1991). Structure of solid tumors and their vasculature: implications for therapy with monoclonal antibodies. *Cancer Cells*, **3**, 77–85.

EVERETT SA, DENNIS MF, TOZER GM, PRISE VE, WARDMAN P AND STRATFORD MRL. (1995). Nitric oxide in biological fluids: analysis of nitrite and nitrate by high preformance ion chromatography. *J Chromatog. A.*, **706**, 437–442.

FEELISCH M. (1991). The biochemical pathways of nitric oxide formation from nitrovasodilators: appropriate choice of exogenous NO donors and aspects of preparation and handling of aqueous NO solutions. *J. Cardiovasc. Pharmacol.*, **17**, S25–S33.

FURCHGOTT RF AND ZAWADZKI JV. (1980). The obligatory role of endothelial cells in the relaxation of arterial smooth muscle by acetylcholine. *Nature*, **288**, 373–376.

HORSMAN MR, CHRISTENSEN KL AND OVERGAARD J. (1992). Relationship between the hydralazine-induced changes in murine tumor blood flow supply and mouse blood pressure. *Int. J. Radiat. Oncol. Bio. Phys.*, **22**, 455–458.

JAIN RK AND WARD-HARTLEY K. (1984). Tumor blood flow–characterisation, modifications and role in hyperthermia. *IEEE Trans.* **SU-31**, 504–526.

KENNOVIN GD, FLITNEY FW AND HIRST DG. (1994). 'Upstream' modification of vasoconstrictor responses in rat epigastric artery supplying an implanted tumour. In *Oxygen Transport to Tissue XV*. Vaupel P (ed.) pp. 411–416. Plenum Press: New York.

LEVICK JR. (1992). *An Introduction to Cardiovascular Physiology*. Butterworth-Heinemann Ltd: Oxford.

MARAGOS CM, WANG JM, HRABIE JA, OPPENHEIM JJ AND KEEFER IK. (1993). Nitric oxide/nucleophile complexes inhibit the *in vitro* proliferation of A375 melanoma cells via nitric oxide release. *Cancer Res.*, **53**, 564–568.

PETERSON H-I. (1991). Modification of tumour blood flow–a review. *Int. J. Radiat. Biol.*, **60**, 201–210.

POSTON L AND TAYLOR PD. (1995). Endothelium-mediated vascular function in insulin-dependent diabetes mellitus. *Clin. Sci.*, **88**, 245–255.

RALEVIC V AND BURNSTOCK G. (1993). *Neural-Endothelial Interactions in the Control of Local Vascular Tone*. RG Landes: Austin, TX, USA.

SAGAR SM, KLASSEN GA, BARCLAY KD AND ALDRICH JE. (1993). Tumour blood flow: measurement and manipulation for therapeutic gain. *Cancer Treat. Rev.*, **19**, 299–349.

STERNBERGH WC AND ADELMAN B. (1992). The temperol relationship between endothelial cell dysfunction and skeletal muscle damage after ischemia and reperfusion. *J. Vasc. Surg.*, **16**, 30–39.

STRATFORD MRL, DENNIS MF, TOZER GM, PRISE VE AND EVERETT SE. (1996). Measurement of nitric oxide in biological materials by high performance ion chromatography and EPR spectroscopy: a comparative study. In *The Biology of Nitric Oxide Part 5*. Moncada S *et al.* (eds) p. 175. Portland Press: London.

THOMSEN LL, LAWTON FG, KNOWLES RG, BEESLEY JE, RIVEROS-MORENO V AND MONCADA S. (1994). Nitric oxide synthase activity in human gynecological cancer. *Cancer Res.*, **54**, 1352–1354.

THOMSEN LL, MILES DW, HAPPERFIELD L, BOBROW LG, KNOWLES RG AND MONCADA S. (1995). Nitric oxide synthase activity in human breast cancer. *Br. J. Cancer*, **72**, 41–44.

TOZER GM, SHAFFI KM, PRISE VE AND CUNNINGHAM VJ. (1994). Characterisation of tumour blood flow using a 'tissue-isolated' preparation. *Br. J. Cancer*, **70**, 1040–1046.

TOZER GM, PRISE VE AND BELL KM. (1995). The influence of nitric oxide on tumour vascular tone. *Acta Oncol.*, **34**, 373–377.

WOOD P, STRATFORD IJ, ADAMS GE, SZABO C, THIEMERMANN C AND VANE JR. (1993). Modification of energy metabolism and radiation response of a murine tumour by changes in nitric oxide availability. *Biochem. Biophys. Res. Commun.*, **192**, 505–510.

WOOD PJ, SANSOM JM, BUTLER SA, STRATFORD IJ, COLE SM, SZABO C, THIEMERMANN C AND ADAMS GE. (1994). Induction of hypoxia in experimental murine tumors by the nitric oxide synthase inhibitor. $N^G$-nitro-L-arginine. *Cancer Res.*, **54**, 6458–6463.

ZYGMUNT PM, HÖGESTÄTT ED AND GRUNDEMAR L. (1994). Light-dependent effects of zinc protoporphyrin IX on endothelium-dependent relaxation resistant to $N\omega$-nitro-L-arginine. *Acta Physiol. Scand.*, **152**, 137–143.